# Application of immunofluorescence-based detection of AFP-L3 in the diagnosis of hepatocellular carcinoma

**DOI:** 10.1097/MD.0000000000042194

**Published:** 2025-04-18

**Authors:** Haibin Shen, Meijin Liu, Die Hu, Fangfang Xie, Qing Jin, Dewang Xiao, Zongbo Peng, Defa Huang

**Affiliations:** a Laboratory Medicine, First Affiliated Hospital of Gannan Medical University, Ganzhou, China; b Laboratory Medicine, People’s Hospital of Ganzhou Economic Development Zone, Ganzhou, China; c The First School of Clinical Medicine, Gannan Medical University, Ganzhou, China.

**Keywords:** AFP, AFP-L3, diagnostic value, hepatocellular carcinoma

## Abstract

Alpha-fetoprotein (AFP) together with Lens culinaris-agglutinin-reactive fraction of AFP (AFP-L3) serve as the preferred tumor markers for hepatocellular carcinoma (HCC) diagnosis. The authors performed diagnostic value analysis on AFP along with AFP-L3 as individual biomarkers and together to determine the optimal biomarker option. Researchers evaluated 149 HCC patient sera and 70 healthy control sera using automatic microchip capillary electrophoresis and liquid phase combination-based analysis. They found the area under the curve value for the conventional marker AFP reached 0.844 and the diagnostic value of AFP-L3 was 0.923. The diagnostic effectiveness did not enhance as both biomarkers were combined for testing. The performance metrics of AFP-L3 increase during disease progression which leads to complete diagnostic sensitivity and perfect specificity and an area under the curve value of 1 during Barcelona clinical liver cancer and D stage.

## 1. Introduction

The digestive system contains liver cancer as its most frequent cancerous tumor which represents the fourth leading global cause of mortality from cancer. According to the Global Cancer Report 2018 by the International Agency for Research on Cancer there were 841,000 liver cancer new cases and 782,000 deaths recorded in 2018.^[1]^ Hepatocellular carcinoma is a major type of primary liver cancer. HCC is related to the tumor stage at the time of diagnosis.^[2]^ The absence of detectable signs and symptoms in early stages causes most hepatocellular carcinomas to reach an advanced stage when patients receive their diagnosis ultimately leading to a negative prognosis. Detecting HCC at an early stage stands as a vital approach to enhance the disease prognosis.

Currently, screening for HCC is based on serum alpha-fetoprotein measurements, imaging techniques, and tissue biopsies.^[3,4]^ The current diagnoses of early-stage HCC remain limited because AFP testing cannot validate atypical results while not detecting all patients along with imaging devices which struggle with small lesions. Research indicates that the diagnostic effectiveness of ultrasound screening for detecting early HCC reaches 45% while combined serum AFP testing only reaches 63% success rate.^[5]^ In addition, although tissue biopsy can accurately diagnose HCC, it is an invasive diagnostic method that may increase needle metastasis.^[6]^ The detection methods for earlier diagnosis of HCC have been expanding to include decarboxylated prothrombin (DCP) alongside glypican-3 and bone bridge protein as potential serum biomarkers. Serum DCP tests diagnosed HCC with a sensitivity range from 48% to 62% and displayed a specificity between 81% and 98% and achieved an accuracy between 59% and 84%.^[7]^ Other recent studies have suggested a potential role for glypican-3 levels in early HCC detection for HCC screening. Liu et al^[8]^ found a significantly higher percentage of gpc3 positivity in early HCC than AFP (76.4% vs 64.3%). The similar result also found at Shang et al^[9]^ study, for early stage HCC, osteopontin had a better sensitivity than AFP (75% vs 46%), but the specificity is lower than AFP (62% vs 93%). Thus HCC demands additional detailed noninvasive diagnostic markers to enhance early diagnosis capabilities.

Clinicians use the lens culinary agglutinin-reactive fraction of fetoprotein subtype as a diagnostic tool because this protein product originates from malignant hepatocytes exclusively.^[10]^ The evaluation of AFP-L3% for detecting early HCC was absent from the previously conducted study which demonstrated a low pooled sensitivity of 0.483 at HCC diagnosis.^[11]^ The main goal of this research was to examine HCC patient related elements impacting AFP-L3 levels and assess its use as a tool for detecting HCC at an early stage.

## 2. Materials and method

### 2.1. Patient samples

The research examined HCC patients diagnosed at the First Affiliated Hospital of Gannan Medical College. The research included pathologically confirmed HCC cases of 149 patients with 70 healthy donors (HD) enrolled throughout January 2021 to December 2022. Clinical symptoms and signs were evaluated through magnetic resonance imaging together with abdominal ultrasound and CT examinations which all patients received. The authors collected clinical information from hospital electronic medical records. Medical professionals performed tumor staging assessments through the Barcelona Clinical Liver Cancer (BCLC) staging system. The study team measured serum AFP levels as well. We established 10 ng/mL as the determining value for AFP measurements. Each patient and all controls provided their informed consent after the physician explanation. The research received permission from the First Affiliated Hospital of Gannan Medical College local ethics committee to proceed.

### 2.2. Sample collection and isolation

The experiment used 6ml of subject whole blood which was drawn into coagulant-promoting tubes with separation gel before serum extraction by centrifugation at 4 °C 1500 g for 15 minutes. The blood serum will be subjected to machine analysis immediatelly or placed at −80 °C for future use.

### 2.3. Immunofluorescence assay for AFP L3

The μTASWako AFP-L3 assay performs immunofluorescence analysis on μTASWako i30 (Osaka, Japan) for the quantitative detection of human serum AFP-L3 ratio together with AFP to assist in malignant tumor diagnosis. The seed extract Lens culinaris-agglutinin functions to bind glycoform AFP-L3 but fails to detect AFP-L1. The reaction towards LCA serves as a basis to achieve separation of total AFP through affinity electrophoresis. The serum samples from 149 patients having Hepatocellular carcinoma and a group of healthy subjects were analyzed using μ TASWako i30 for their AFP and AFP L3 levels.

### 2.4. Statistical analysis

The study uses mean ± SE as its quantitative data presentation method. The statistical analysis occurred using software Graphpad prism 8.0. The comparison between 2 groups used Student *t* test and 1-way ANOVA for 3 or more group analysis. The statistical analysis for receiver operating characteristic (ROC) curve assessment occurred through version 18.0 of MedCalc software. *P* < .05was considered to be statistically significant.

## 3. Results

A total of 219 cases in this study consisted of 149 HCC patients and 70 HD (Table S1, Supplemental Digital Content, http://links.lww.com/MD/O680 and Table S2, Supplemental Digital Content, http://links.lww.com/MD/O681, raw data for each).

### 3.1. Associations between AFP-L3 levels and clinical characteristics of HCC patients

The relationship between AFP-L3 levels and clinical characteristics in HCC patients are shown in Table 1. AFP-L3 levels were not statistically different with age, gender, HBV infection and liver cirrhosis (*P* = .132, *P* = .139, *P* = .175, and *P* = .525). However, tumor size, number of tumors, portal vein tumor thrombus (PVTT) and BCLC staging were statistically different from AFP-L3 (*P* < .0001, *P* = .0001, *P* < .0001, *P* < .0001, and *P* < .0001). For the BCLC stage, the levels of AFP-L3 were 0.852 ± 3.366, 1.618 ± 2.650, 3.293 ± 3.176, and 13.212 ± 10.686 for BCLC-A, BCLC-B, BCLC-C, and BCLC-D stages, respectively, showing a significant increasing trend.

**Table 1:** Associations between plasma AFP-L3 (%) level and clinical characteristics of HCC patients.

Clinico-pathological characteristics		N (149)	AFP-L3	*P*
Age (yr)	<50	36	3.309 ± 7.14	.132
	≥50	113	1.857 ± 3.915	
Gender	Female	25	1.166 ± 2.167	.139
	Male	124	3.199 ± 6.683	
HBV infection	Absent	76	2.741 ± 6.510	.175
	Present	73	1.633 ± 2.499	
Liver cirrhosis	Absent	61	2.511 ± 6.282	.525
	Present	88	1.981 ± 3.859	
Tumor size	<5 cm	110	0.963 ± 2.785	<.0001
	≥5 cm	48	5.680 ± 7.588	
Tumor number	Single	100	0.972 ± 3.000	<.0001
	Multiple	49	4.700 ± 6.960	
PVTT	Absent	129	1.374 ± 3.226	<.0001
	Present	20	7.511 ± 9.431	
BCLC stage	A	67	0.852 ± 3.366	<.0001
	B	60	1.618 ± 2.650	
	C	12	3.293 ± 3.176	
	D	9	13.212 ± 10.686	

AFP = alpha-fetoprotein, BCLC = Barcelona clinical liver cancer, HCC = hepatocellular carcinoma, PVTT = portal vein tumor thrombus.

### 3.2. The diagnostic efficiency of AFP-L3 for determination of HCC

**Figure 1.**
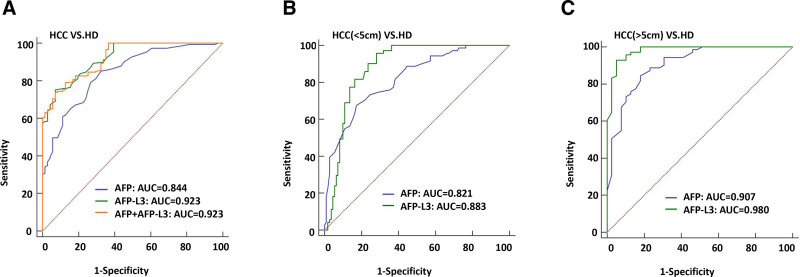
The ROC curve analysis the diagnosis efficiency of AFP and AFP-L3 for HCC. (A) The diagnostic ability to distinguish HCC patients from HD. (B) The diagnostic ability to distinguish HCC patients with tumor size <5 cm from HD. (C) The diagnostic ability to distinguish HCC patients with tumor size greater than or equal to 5 cm from HD. AFP = alpha-fetoprotein, HCC = hepatocellular carcinoma, HD = healthy donors, ROC = receiver operating characteristic.

**Figure 2.**
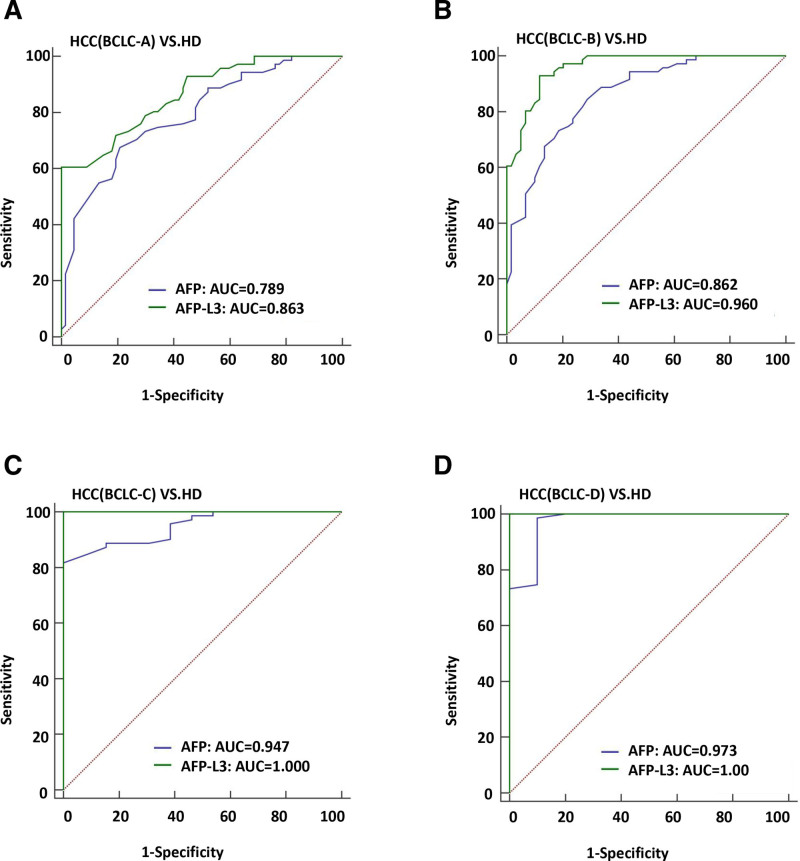
The ROC curve analysis the diagnosis efficiency of AFP and AFP-L3 for BCLC stage in HCC patients. (A) The diagnostic ability to distinguish HCC patients with BCLC-A stage from HD. (B) The diagnostic ability to distinguish HCC patients with BCLC-B stage from HD. (C) The diagnostic ability to distinguish HCC patients with BCLC-C stage from HD. (D) The diagnostic ability to distinguish HCC patients with BCLC-D stage from HD. AFP = alpha-fetoprotein, BCLC = Barcelona clinical liver cancer, HCC = hepatocellular carcinoma, HD = healthy donors, ROC = receiver operating characteristic.

The ROC curve analysis was conducted to assess the diagnostic efficiency of AFP and AFP-L3 in determining HCC. The sensitivity of AFP for HCC was 85.23%, the specificity was 67.61%, and the ROC was 0.844 (Fig. 1A and Table 2). The sensitivity of AFP-L3 for HCC was 75.17%, the specificity was 92.96%, and the ROC was 0.923 (Fig. 1A and Table 2). The combined use of AFP and AFPL-3 did not improve the diagnostic performance (Fig. 1A and Table 2). In addition, the diagnostic performance of AFPL-3 improved as the tumor grew larger. For HCC tumors smaller than 5cm, the sensitivity of AFP-L3 was 95.77%, the specificity was 71.82%, and the ROC was 0.883 (Fig. 1B and Table 2). When the tumor was larger than 5cm, the sensitivity of AFP-L3 was 92.96%, the specificity was 94.87, and the ROC was 0.98 (Fig. 1C and Table 2). In addition, the diagnostic performance of AFP-L3 increased with the severity of disease (Fig. 2A–D and Table 2). When HCC was in BCLC-D stage, the sensitivity and specificity of AFP-L3 diagnosis were 100% and ROC was 1 (Fig. 2D and Table 2).

**Table 2:** Main parameters of ROC curve analysis results.

Variable	AUC	95% CI	Sensitivity (%)	Specificity (%)	Youden index	*P*
HCC-HD						
AFP	0.844	0.789 to 0.889	85.23	67.61	0.528	<.0001
AFP-L3	0.923	0.879 to 0.954	75.17	92.96	0.681	<.0001
AFP + AFP-L3	0.923	0.880 to 0.955	73.83	92.96	0.668	<.0001
HCC (<5 cm)-HD						
AFP	0.821	0.758 to 0.874	67.61	82.73	0.500	<.0001
AFP-L3	0.883	0.827 to 0.926	95.77	71.82	0.675	<.0001
HCC (≥5cm)-HD						
AFP	0.907	0.837 to 0.954	84.51	82.05	0.665	<.0001
AFP-L3	0.980	0.933 to 0.997	92.96	94.87	0.878	<.0001
HCC (BCLC-A)-HD						
AFP	0.789	0.711 to 0.854	67.61	79.10	0.467	<.0001
AFP-L3	0.863	0.795 to 0.916	60.56	100	0.605	<.0001
HCC (BCLC-B)-HD						
AFP	0.862	0.790 to 0.916	84.51	71.19	0.5569	<.0001
AFP-L3	0.960	0.911 to 0.987	92.96	88.14	0.810	<.0001
HCC (BCLC-C)-HD						
AFP	0.947	0.875 to 0.984	81.69	100	0.897	<.0001
AFP-L3	1	0.957 to 1.000	100	100	1	<.0001
HCC (BCLC-D)-HD						
AFP	0.973	0.911 to 0.996	98.59	99.00	0.8859	<.0001
AFP-L	1	0.955 to 1.000	100	100	1	<.0001

AFP = alpha-fetoprotein, AUC = area under the curve, BCLC = Barcelona clinical liver cancer, CI = confidence interval, HCC = hepatocellular carcinoma, HD = healthy donots, PVTT = portal vein tumor thrombus, ROC = receiver operating characteristic.

## 4. Discussion

The early detection of HCC is vital because it enables curative interventions for improving patient survival outlooks and prognosis.^[12,13]^ The search for meaningful early diagnosing biomarkers represents a matter of great importance. We designed this case–control investigation to determine the utilities of new diagnostic evaluation methods.

The early stages of HCC show minimal symptoms which leads most patients to seek hospital care only after experiencing liver pain and jaundice symptoms. The majority of these patients reach late stages by this point because conventional surgery becomes impossible thus reducing their chance of recovery.^[14]^ The prognosis of patients improves significantly when healthcare teams perform accurate early diagnosis of HCC. The diagnostic gold standard for HCC remains pathologic examination which proves harmful to patients. MRI scanning can identify lesions promptly yet its detection depends largely on interpretation by doctors who might wrongly identify the results. The detection method of tumor markers provides essential diagnostic abilities which healthcare institutions utilize widely across tumor diagnosis processes. The use of serological biomarkers represents an appealing approach to support hepatocellular carcinoma surveillance systems and early diagnosis which also benefits monitoring of hepatocellular carcinoma therapy.^[15,16]^ The diagnosis and monitoring of hepatocellular carcinoma significantly depends on alpha-fetoprotein (AFP) because it stands as the leading biomarker among those currently known. The levels of serum AFP showed insufficient effectiveness in terms of both positive and negative detection results. Several studies revealed that HCC detection in HCV patients through AFP testing achieves a positive rate of 41% to 65% and negative detection rate of 80% to 94% separately.^[17]^ In addition, AFP levels may be elevated in patients with nonspecific diseases such as cirrhosis or chronic HCV exacerbation.^[18,19]^ The medical research community has studied 2 additional serum biomarkers for hepatocellular carcinoma known as prothrombin induced by vitamin K absence-II (PIVKA-II).^[20]^ The main objective of this research investigated the diagnostic power of Lens culinaris-agglutinin-reactive fraction of AFP in HCC detection at its early stages or when AFP results come back negative.^[21]^ The Lens culinaris-agglutinin A reactivity property indicates that AFP-L3 represents a fucosylated form of AFP. Scientists use AFP-L3 to separate AFP elevations that result from HCC from those that originate from benign liver disease.^[22]^ The AFP-L3 isoform achieves higher diagnostic specificity for HCC since it measures against the total AFP concentration.^[23]^

The research revealed that HCC patients with elevated AFP-L3 demonstrated associations with tumor dimensions, tumor number and PVTT and BCLC staging yet separate from age, gender, HBV infection and liver cirrhosis groups. The diagnostic performance evaluation showed AFP as the traditional marker yielded 0.844 area under the curve (AUC) but AFP-L3 reached an AUC of 0.923. The diagnostic value of combining AFP with AFP-L3 failed to enhance in testing patients. When cancers progress to BCLC-D stage the diagnostic sensitivity along with specificity of AFP-L3 reaches 100% while the AUC reaches value 1. The study proposes AFP-L3 as an optimal marker for HCC detection because it provides precise diagnostic standards for HCC evaluation.

The research contains several constraints which need Acknowledgments. The conducted study operated from a single center while working with a limited patient sample that exhibited different disease reasons between participants. For complete clinical assessment of AFP-L3’s value in HCC diagnosis additional research must focus on combined multicenter research projects and growing patient case numbers.

In conclusion, AFP-L3 detection appears to provide a valuable diagnostic tool for HCC identification at an early stage thus making it suitable for medical application.

## Author contributions

**Conceptualization:** Dewang Xiao, Zongbo Peng.

**Funding acquisition**: Defa Huang.

**Investigation:** Die Hu, Fangfang Xie, Qing Jin.

**Project administration:** Defa Huang.

**Writing – original draft:** Haibin Shen.

**Writing – review & editing:** Meijin Liu.

## Supplementary Material


